# Glycated haemoglobin (HbA_1c_) for postmortem diagnosis of diabetes

**DOI:** 10.1080/20961790.2018.1452354

**Published:** 2018-04-18

**Authors:** Delia Lepik, Mailis Tõnisson, Anne Kuudeberg, Marika Väli

**Affiliations:** a Department of Pathological Anatomy and Forensic Medicine, Faculty of Medicine, Institute of Biomedicine and Translational Medicine, University of Tartu, Tartu,Estonia; b Department of Internal Medicine, Faculty of Medicine, Institute of Clinical Medicine, University of Tartu, Tartu, Estonia; c Southern Estonian Forensic Medical Examination Department, Estonian Forensic Science Institute, Tallinn, Estonia; d Management Board, Estonian Forensic Science Institute, Tallinn, Estonia

**Keywords:** Forensic science, forensic pathology, postmortem, diabetes, glycated haemoglobin

## Abstract

The study was conducted at the Estonian Forensic Science Institute in 2008–2014 as continuous part of our previous study of alcohol and premature death in Estonian men. Autopsy data from 504 cases of male deaths (ages 19–79) were collected and blood and urine samples for glycated haemoglobin (HbA_1c_), liver enzymes and alcohol concentration were analysed. The aim of our research was to find undiagnosed diabetes and diabetes risk cases postmortem on the basis of increased values of HbA_1c_. HbA_1c_ was within the reference value 4.8%–5.9% (29–42 mmol/mol), in 88.1% (*n* = 444) of cases, below reference value in 2.4% (*n* = 12), in the risk group of diabetes, HbA_1c_ 6.0%–6.4% (42–46 mmol/mol) was within 5.8% (*n* = 29), and HbA_1c_ result of ≥6.5% (48 mmol/mol) manifested in 3.8% (*n* = 19) of cases. The higher the age, the more cases with HbA_1c_ value ≥6.0% (42 mmol/mol) occurred. In the group of external causes of death (*n* = 348), the HbA_1c_ value of ≥6.5% (48 mmol/mol) HbA_1c_ occurred in four cases. The HbA_1c_ value was ≥6.5% (48 mmol/mol) in 78.9% of 156 cases when the cause of death was disease, of which 58% were cardiovascular diseases. The prevalence of diabetes and diabetes risk was found lower compared to population-based study, as majority of the deceased were young and middle-aged males and no females were included. In the case of poisoning with narcotic substances, HbA_1c_ was within the reference range. A negative correlation occurred between alcohol intoxication and HbA_1c_ value. A positive correlation between ALT and HbA_1c_ was found – the higher stage of liver damage correlated with the higher HbA_1c_ level.

## Introduction

Postmortem diagnosis of glucose metabolism disorders include acute complications of diabetes mellitus as causes of death can be difficult and vague because of the lack of characteristic morphological findings. Diabetes mellitus is one of the most common chronic diseases in many countries, and its occurrence continues to increase. According to International Diabetes Federation data there were 425 million people aged of 20–79 years with diabetes in the world in 2017, and one of two adults with diabetes are undiagnosed (212 million). In Europe, the number of people with diabetes was estimated to be 58 million in 2017 [1].

Studies on alcohol consumption, pre-diabetes and type 2 diabetes risks have different and inconsistent results, showing either a protective or harmful effect or a more complex relationship. Data from epidemiological studies indicate a protective effect of moderate alcohol consumption. No beneficial effect can be attributed to high alcohol consumption [2,3]. Many studies showed that heavy alcohol consumption is a major cause of chronic pancreatitis and a risk factor for type 2 diabetes mellitus and pancreatic cancer [4]. Influence may also depend on specific alcoholic beverages. High consumption of spirits and beer seemed to increase the risk of pre-diabetes and type 2 diabetes but high wine intake showed a reduced risk of pre-diabetes [5]. The persons having impaired fasting glucose (fasting serum glucose 6.1–6.9 mmol/L and 2 h glucose <7.8 mmol/L) or impaired glucose tolerance (fasting serum glucose <6.1 mmol/L and 2 h glucose 7.8–11.0 mmol/L) were referred to as pre-diabetes cases [5].

For diagnosing diabetes blood glucose should be determined, but as the blood glucose content decreases postmortem because of continuing metabolism, it is not practical to determine blood glucose. Glucose metabolism may be evaluated on the basis of the results of glycated haemoglobin which are not strongly affected by glucose metabolism postmortem [6–8]. The test plays a critical role in the management of a patient with diabetes, since it correlates well with both microvascular and, to a lesser extent, macrovascular complications and is widely used as the standard biomarker for the adequacy of glycaemic management [9]. The amount of HbA_1c_ in the blood is dependent on mean glucose levels present during the 8–12 weeks preceding measurement, as HbA_1c_ accumulates in red blood cells during their 120-day lifespan, and therefore it is useful for long-term blood glucose control in diabetics [10–12]. For revealing undiagnosed or poorly managed diabetes in a post-mortem context, HbA_1c_ levels are useful for distinguishing diabetic ketoacidosis from starvation or alcoholic ketoacidosis, reveal death caused by hyperosmolar hyperglycaemic state [6,7,13–16] and diagnose fatal diabetic coma [17]. HbA_1c_ is relatively stable for analysing postmortem samples [18–20] and correlates well with clinical samples [19]. Ensuring the diagnosis of diabetes mellitus by morphological findings is very difficult and in many cases it remains undiagnosed. Therefore, it is important to determine if HbA_1c_ values are affected by other concomitant diseases in addition to diabetes and if it is associated with cause of death due to external causes (injury).

The first aim of our research was to find undiagnosed diabetes cases and diabetes risk cases postmortem on the basis of increased values of glycated haemoglobin. The second aim was to find changes in the values of liver enzymes, alanine aminotransferase (ALT), aspartate aminotransferase (AST) and gamma glutamyl transferase (GGT), and the association between liver enzymes and glycated haemoglobin in chronic alcohol abusers in addition to liver damage diagnosed on the basis of macroscopic findings and to find out cases of possible secondary diabetes.

## Materials and methods

The study was conducted at the Estonian Forensic Science Institute in 2008–2014 as continuous part of our previous study of alcohol and premature death in Estonian men. The study target population was all deaths in Estonia subject to forensic autopsy of men aged over 18 years. The criterion for inclusion into the present study was possibility to obtain vitreous humour and blood. At autopsy, blood was collected from peripheral vessels, usually from femoral vein, with 20 mL syringe and 14-gauge needle. Blood was collected into 10 mL vacutainer tubes – in an EDTA tube for glycated haemoglobin and one plain vacutainer for liver enzymes and with sodium fluoride for blood alcohol concentration. Urine was taken directly from the bladder with 20 mL syringe and 14-gauge needle. There was also one plain tube for urine. Urine samples were collected for alcohol and narcotics. Vitreous humour was aspirated from both eyes using a syringe.

The samples were refrigerated at 4 °C in the Forensic Science Departments of the Estonian Forensic Science Institute (EFSI) and dispatched at the same temperature conditions to the Southern Estonian Department within 24 h. For the determination of liver enzymes, blood was centrifuged at 2 100 r/min for 15 min in Eppendorf cooled centrifuge 4 °C and thereafter the serum was removed. If it was not possible to get serum, this case was excluded from the study. Data from the remaining 504 cases was used in the analysis. EDTA-blood for the determination of HbA_1c_ and serum for the determination of liver enzymes were sent to the United Laboratories of the Tartu University Hospital. Vitreous humour was used for other studies. ALT and AST levels were measured using the kinetic photometric method as following: ALT and AST according to International Federation of Clinical Chemistry and Laboratory Medicine (IFCC) with pyridoxal phosphate activation (Cobas 6000, Roche) – *in vitro* tests for the quantitative determination of ALT and AST with pyridoxal phosphate activation in human serum and plasma on Roche/Hitachi cobas c systems. Both tests’ measuring range is 5–700 U/L (0.08–11.7 μkat/L) and lower detection limit 5 U/L (0.08 μkat/L), and precision was determined using human samples and controls in an internal protocol with repeatability (*n* = 21) and intermediate precision (3 aliquots per run, 1 run per day, 20 days). The consensus measuring ranges (IFCC) for ALT and AST are <50 U/L in men and <35 U/L in women. GGT levels were measured with the kinetic colorimetric method as in the following: γ-Glutamyltransferase ver.2 Standardized against IFCC/Szasz (Cobas 6000, Roche) – *in vitro* test for the quantitative determination of γ-glutamyltransferase (GGT) in human serum and plasma on Roche/Hitachi cobas c systems. Measuring range is 3–1 200 U/L (0.05–20.0 μkat/L) and lower detection limit of the test 3 U/L (0.05 μkat/L), and precision was determined using human samples and controls in an internal protocol with repeatability (*n* = 21) and intermediate precision (3 aliquots per run, 1 run per day, 21 days). The consensus measuring ranges (IFCC) for GGT are <60 U/L in men and <40 U/L in women. HbA_1c_ was measured using the Tina-quant Hemoglobin A1c Gen.3 – Hemolysate Application – Standardized according to IFCC transferable to Diabetes Control and Complications Trial/National Glycohemoglobin Standardization Program (DCCT/NGSP) (Cobas Integra 400 plus, Roche) – *in vitro* test for the quantitative determination of mmol/mol of hemoglobin A1c (IFCC) and percentage of hemoglobin A1c (DCCT/NGSP) in hemolysate prepared from whole blood on Roche clinical chemistry analysers. The HbA_1c_ determination is based on the turbidimetric inhibition immunoassay (TINIA) for hemolyzed whole blood. The final result is expressed as mmol/mol or percentage of HbA_1c_ and is calculated from the HbA_1c_/Hb ratio. Measuring range of 23–196 mmol/mol HbA_1c_ (IFCC) and 4.2%–20.1% HbA_1c_ (DCCT/NGSP) at a typical haemoglobin concentration of 8.2 mmol/L (13.2 g/dL). Lower limits of measurement: accordingly limit of blank and limit of detection for Hb 0.31 mmol/L and 0.62 mmol/L and for HbA_1c_ 0.12 mmol/L and 0.18 mmol/L. Repeatability and intermediate precision were determined using human samples and controls in accordance with the Clinical and Laboratory Standards Institute (CLSI) EP5 requirements (2 aliquots per run, 2 runs per day, 21 days).

For the assessment of laboratory results, Tartu University Hospital’s United Laboratories reference values for patients/living persons were used. The reference values for live persons are as follows: ALT <41 U/L, AST <38 U/L and GGT <61 U/L [20]. The reference values for HbA_1c_ are as follows: normal value 4.8%–5.9% (by the IFCC 29–42 mmol/mol), below reference value <4.8% (<29 mmol/mol), risk group for diabetes 6.0%–6.4% (42–46 mmol/mol), diabetes mellitus ≥6.5% (≥48 mmol/mol) [21].

Urine and blood alcohol concentrations (BAC) were measured at the Chemistry Laboratory of the EFSI using headspace gas chromatography. Urine samples were screened in EFSI for narcotics, using a rapid urine test (Multiscreen 10 MTD; Biomedical Diagnostics) for measure of amphetamine, methamphetamine, cocaine, ecstasy, morphine, methadone, cannabis metabolite Δ-tetrahydrocannabinole, benzodiazepines, barbiturates and tricyclic antidepressants. Where screening results were positive, further confirmatory analysis was undertaken in EFSI using combined gas chromatography and mass spectrometry.

For each case a study record was completed to which the record number, the EFSI Department, the autopsy number, and the deceased's date of birth, age, date of death and date of autopsy were entered using general data. Of more specific data, macroscopic, microscopic and laboratory findings (ethanol concentration, concentration of alcohol surrogates, illegal and legal drugs, liver enzymes and HbA_1c_) were documented retrospectively. Cause of death according to ICD code, manner of death, main disease and concomitant diseases were added to the record summary. The causes of death were classified on morphological and toxicological bases. The data were analysed by the following age groups: ≤29 years, 30–39 years, 40–49 years, 50–59 years and ≥60 years.

Statistical analysis was performed using the Statistica 13.0 statistical program. Spearman rank order correlation (denoted as *r_s_*), multiple response items and factorial dispersion analysis were used to assess bivariate relationships. The descriptive statistics were used to analyse cases’ general data. A *P*-value <0.05 was considered statistically significant.

## Results

Autopsy data from 504 cases of male deaths (ages 19–79) were collected and samples were analysed. HbA_1c_ was within the reference value 4.8%–5.9% (29–42 mmol/mol) in 88.1% (*n* = 444) of cases, HbA_1c_ was below reference value in 2.4% (*n* = 12), in the risk group of diabetes, HbA_1c_ 6.0%–6.4% (42–46 mmol/mol) was within 5.8% (*n* = 29), and HbA_1c_ result of ≥6.5% (48 mmol/mol) manifested in 3.8% (*n* = 19) of cases. Previously was known to have diabetes in five males.

### Age and HbA_1c_ values

Eighty-five per cent of the deceased were aged 30–60 years. The largest age group was 40–49 years old, 38.1% of all investigated deaths. The higher the age, the more cases with HbA_1c_ value of >6.0% (42 mmol/mol) occurred (*r_s_* = 0.2, *P* < 0.05). In an age group ≤29 years there was only one case where the HbA_1c_ value was ≥6.5% (48 mmol/mol) (a 26-year-old youth who died of gastrointestinal haemorrhage and who had alcoholic liver steatosis as the main disease. There was a previous history of diabetes). In the 30–39 age group there were six cases with a HbA_1c_ value of > 6.0% (42 mmol/mol) and one case with a HbA_1c_ value of ≥6.5% (48 mmol/mol); in the 40–49 age group there were 12 cases with a HbA_1c_ value of > 6.0% (42 mmol/mol) and 4 cases with a HbA_1c_ value of ≥6.5% (48 mmol/mol); in the 50–59 age group there were 11 HbA_1c_ values of > 6.0% (42 mmol/mol) and 11 values of ≥6.5% (48 mmol/mol) (that is 45.8% of all values of > 6.0% (42 mmol/mol)); in the ≥60 age group there were two cases with a HbA_1c_ value of ≥6.5% (48 mmol/mol). The age distribution of subjects and HbA_1c_ values are presented in Table 1.

**Table 1.** The age distribution of subjects and HbA_1c_ values.

Age group (years)	*N*	<4.8%	4.8%–5.9%	6.0%–6.4%	≥6.5%
**≤**29	66	1 (1.5%)	64 (96.7%)	0	1 (1.5%)
30–39	113	2 (1.8%)	104 (92.0%)	6 (5.3%)	1 (0.9%)
40–49	192	6 (3.1%)	170 (88.5%)	12 (6.3%)	4 (2.1%)
50–59	124	3 (2.4%)	99 (79.8%)	11 (8.9%)	11 (8.9%)
≥60	9	0	7 (77.8%)	0	2 (22.2%)
Total	504	12 (2.4%)	444 (88.1%)	29 (5.8%)	19 (3.8%)

### Cause of death and HbA_1c_ values

On the basis of the cause of death we established the following study groups: (1) external causes of death (*n* = 348): injury, other external causes (hypothermia, electrocution), drowning, hanging, other asphyxias (e.g. asphyxia with foreign bodies, inhalation of gastric contents), alcohol poisoning, illegal drug poisoning, fire fatality (e.g. carbon monoxide poisoning, burns of multiples body regions), (2) diseases (*n* = 156): cardiovascular diseases (chronic ischaemic heart disease, myocardial infarction, alcoholic and hypertrophic cardiomyopathy, hypertensive diseases), alcoholic liver disease and other diseases (pneumonia, haemorrhagic gastritis). Cause of death, age and levels of HbA_1c_, ALT, AST, GGT and BAC are presented in Table 2.

**Table 2.** Cause of death, age and levels of HbA_1c_, ALT, AST, GGT and BAC.

		Age (years)		HbA_1c_ (% (mmol/mol))		ALT (U/L)		AST (U/L)		GGT (U/L)		BAC (mg/g)	
Cause of death	*n*	Range	Median	Range	Median	Range	Median	Range	Median	Range	Median	Range	Median
Injury	82	21–54	39.5	4.8–6.4 (29–46)	5.4 (36)	35–28 900	689	99–19 850	983.5	15–1 455	58	0–4.3	2.0
Other external causes	19	32–58	45.0	4.7–6.2 (28–44)	5.5 (37)	51–9 374	273	82–5 717	814	26–947	59.5	0–3.2	0.0
Drowning	26	26–54	41.0	4.8–5.9 (29–42)	5.4 (36)	32–4 792	268	172–2 039	747	21–348	90	0–3.9	2.3
Hanging	91	19–68	44.0	4.9–8.4 (30–68)	5.5 (37)	57–439 500	1 342	134–195 200	1 112	21–327	76.5	0–4.0	1.4
Others asphyxias	34	20–66	41.0	5.0–6.6 (31–47)	5.5 (37)	112–5 919	639	156–6 208	897	30–1 608	124	0–6.3	1.5
Fatal alcohol abuse	43	29–54	46.0	4.8–6.9 (29–52)	5.4 (36)	40–17 000	483	63–9 984	945	29–725	124	0–6.6	3.9
Drug intoxication	36	25–43	28.0	4.5–5.9 (26–42)	5.3 (34)	165–42 580	1 833	287–24 540	1 664	34–1 102	123	0–2.7	0.0
Fire fatality	17	26–58	46.0	4.3–5.7 (24–39)	5.3 (34)	45–29 898	745	116–17 653	613	34–1 102	123	0–4.4	2.7
Cardiovascular diseases	87	31–79	47.0	4.3–10.7 (24–93)	5.6 (38)	29–8 363	906	122–9 817	1 026	20–1 884	89	0–3.8	0.0
Alcoholic liver disease	35	26–60	48.0	4.5–6.9 (26–52)	5.5 (37)	101–13 793	1 078	237–13 050	1 847	47–988	340	0–2.6	0.0
Others diseases	34	29–54	47.0	4.7–9.9 (28–85)	5.6 (38)	49–11 172	771.5	106–9 596	1 266.5	23–900	82	0–2.8	0.0

BAC: blood alcohol concentration.

In the group of external causes of death, an HbA_1c_ value of ≥6.5% (48 mmol/mol) HbA_1c_ occurred in four cases (1.1%): in two cases the cause of death was mechanical asphyxia (hanging), in one case other asphyxiation (aspiration of vomit) and in one case ethanol poisoning. In the case of acute alcohol poisoning the HbA_1c_ value was 4.8%–6.9% (29–52 mmol/mol) (median 5.4% (36 mmol/mol)) and in the case of poisoning with narcotic substances, and in the injury and hanging groups, HbA_1c_ was within the reference range (median 5.4% (36 mmol/mol)). A negative correlation occurred between alcohol intoxication and HbA_1c_ value (*r_s_* = −0.1, *P* < 0.05). In the whole study group 54.6% of the deceased ethanol was found in blood.

In the diseases group, in 78.9% (*n* = 123) of cases the HbA_1c_ value was ≥6.5% (48 mmol/mol), of which 57.9% were cardiovascular diseases. In the alcoholic liver damage group, the HbA_1c_ of value ≥6.5% (48 mmol/mol) occurred in two cases. In previously known diabetes patients (*n* = 5) HbA_1c_ values were as in the following: 9.9% (85 mmol/mol) in male whose cause of death was ischaemic heart disease, 10.7% (93 mmol/mol) in male with myocardial infarction, 5.6% (38 mmol/mol) in male with other unspecified heart disease, and 6.2% (44 mmol/mol) and 9.44% (78 mmol/mol) in two cases with pneumonia. On the basis of forensic autopsy findings diabetes was diagnosed only in one case where it was previously undiagnosed and where the direct cause of death was myocardial infarction; the biochemical characteristics were: HbA_1c_ 8.93% (74 mmol/mol), ALT 2 415 U/L, AST 1 439 U/L and GGT 28 U/L.

### Comorbidities and HbA_1c_ values

Cardiovascular diseases occurred in 75% of all deceased, of which 47.2% (*n* = 238) were atherosclerosis of the aorta and coronary arteries, 4.8% (*n* = 24) hypertension, 3.2% (*n* = 16) alcoholic cardiomyopathy, 3.2% (*n* = 16) dilating cardiomyopathy, 4.5% (*n* = 23) hypertrophic cardiomyopathy, 11.7% (*n* = 59) chronic ischaemic heart disease and 0.4% (*n* = 2) other cardiovascular diseases (valve defect, myocarditis). Of the cases, 19.8% (*n* = 100) were without concurrent cardiovascular pathology and in 26 cases no data were reported about cardiovascular pathology. Of the deceased with HbA_1c_ value ≥6.0% (42 mmol/mol), 33.4% (*n* = 15) had ischaemic heart disease as well as atherosclerosis of the aorta and coronary arteries, 11.1% (*n* = 5) had hypertrophic cardiomyopathy and other pathologies occurred as single cases. In two cases with an HbA_1c_ value ≥6.0% (42 mmol/mol) no cardiovascular diseases occurred.

Liver diseases occurred in 58% of subjects, of which focal steatosis made up 22.6% (*n* = 114), diffuse steatosis 25.8% (*n* = 130), incomplete fibrosis 2.4% (*n* = 12), incomplete cirrhosis 3.8% (*n* = 19) and alcoholic liver damage 3.6% (*n* = 18). Without liver pathology were 34.9% (*n* = 176) and in 35 cases had no microscopically data. With an HbA_1c_ value ≥6.0 (42 mmol/mol) there was focal liver steatosis of 35.6%, diffuse steatosis of 28.9%, cirrhosis of 4.5% and alcoholic liver damage of 2.2%. Of these, 24.2% had no liver pathology (in other cases the microscopically pathology were not determined).

Pancreatic diseases were found in 14.5% (*n* = 73), of which there were 5 cases of acute pancreatitis and 68 cases of chronic pancreatitis; in 65.9% (*n* = 332) cases no pathology occurred (in 99 cases it was not possible to determine pancreatic pathology). In subjects with HbA_1c_ value ≥6.0% (42 mmol/mol) acute pancreatitis occurred in 2.2% (*n* = 3) and chronic pancreatitis in 15.6% (*n* = 7), no pancreatic pathology occurred in 64.4% of the cases (in other cases the microscopically pathology were not determined).

In the case of mechanical asphyxiation, atherosclerosis of the aorta and coronary arteries was diagnosed as a concomitant disease, in one case hypertension and liver lipidosis; in the case of other asphyxiation the concomitant diseases were dilating cardiomyopathy and liver lipidosis; and in the case of alcohol poisoning ischaemic heart disease was diagnosed as the concomitant disease.

The positive correlation between ALT and HbA_1c_ (*r_s_* = 0.139 3, *P* < 0.05) was found: the higher the stage of damage the higher the HbA_1c_ level. High GGT values occurred in the liver alcoholic damage group. Liver alcoholic damage occurred in 35 cases with a GGT activity median of 340 U/L. The value of the GGT value was also significantly higher than in other cause of death groups (*P* < 0.05). The increase of GGT activity correlates with the risk of death due to atherosclerosis of the coronary arteries and other heart diseases. Table 3 shows the association of liver pathology with HbA_1c_ value and ALT, AST and GGT.

**Table 3.** The association of liver pathology with HbA_1c_ value and ALT, AST and GGT mean value.

Liver pathology	Hb1Ac (% (mmol/mol))	ALT (U/L) Mean	AST (U/L) Mean	GGT (U/L) Mean
Without pathology	4.8–5.9 (29*–*42)	2 802.1	2 075.9	79.6
	6.0–6.4 (42–46)	6 333.4	4 510.4	86.0
	>6.4 (46)	1 264.0	1 375.0	47.5
Focal steatosis	4.8–5.9 (29*–*42)	8 321.0	4 663.4	121.3
	6.0–6.4 (42–46)	4 009.0	2 788.3	100.3
	>6.4 (46)	3 449.6	1 590.6	126.0
Diffuse steatosis	<4.8 (29)	1 173.5	2 895.5	475.0
	4.8–5.9 (29*–*42)	2 382.8	2 837.8	277.5
	6.0–6.4 (42–46)	1 797.8	1 554.9	146.5
	>6.4 (46)	1 997.3	3 643.0	178.0
Fibrosis	4.8–5.9 (29–42)	1 350.1	1 513.3	279.0
Cirrhosis	<4.8 (29)	203.0	931.0	787.5
	4.8–5.9 (29*–*42)	3 186.7	4 614.8	280.7
	6.0–6.4 (42–46)	4 687.0	2 384.0	199.0
	>6.4 (46)	72.0	151.0	410.0
Alcoholic liver damage	<4.8 (29)	420.0	1 562.0	947.0
	4.8–5.9 (29–42)	779.1	1 338.5	284.3
	6.0–6.4 (42–46)	247.0	909.0	352.0

## Discussion

The postmortem determination of complications of diabetes mellitus is difficult depending on the duration of disease. The changes as pancreas islet loss, atherosclerosis, microangiopathy, retinopathy, nephropathy and neuropathy are non-specific signs. Qualitative tests with stripes and tablets (glucose, bilirubin or acetone) or also by using electronic test devices may help to confirm or exclude certain suspected diagnoses [17]. In one study it was found that the calvariae of the diabetics showed reduced brightness and degree of the darker colour was correlated with the duration of illness [22]. The other researchers did not detect colour differences between diabetics and non-diabetics, but found correlation between yellowish bone colour and the subjects’ age [23]. The recent study has shown significant correlation of bone yellowing with HbA_1c_ and age [24] and it is suggested that it is due to accumulation of advanced glycation end products produced in the Maillard or browning reaction by non-enzymatic means [25]. Histological examinations can reveal diabetic glomerulosclerosis (Kimmelstiel Wilson syndrome or nodular glomerulosclerosis) due to angiopathy of capillaries of renal cortex and glycogen nephrosis in cases long-lasting high hyperglycaemia (Armanni-Ebstein cells in straight sections of the proximal tubules) [26,27]. Biochemical analyses, including blood glycated haemoglobin, fructosamin [8,20,28] and anhydroglucitol [29,30], determination, may complement postmortem investigations and provide useful information for determining the cause of death even in corpses with advanced decomposition. In post-mortem specimens, HbA_1c_ was found stable from 4 to 36 h [18] or for at least 72 h (72 or more hours following death) [19,20], and EDTA was found to be preferable preservative, as samples can be measured after four weeks storage at 4 °C [13]. But interpretation of biochemical analyses is difficult because of postmortem blood alterations involving glucose metabolic pathways. For comparison of the clinical and postmortem results, the measurement of blood glycated haemoglobin was chosen because fructosamin or anhydroglucitol is not used for glycaemic control in clinical laboratories in Estonia.

In Estonia, the adults (20–79 years old) with diabetes were estimated to be 55 000 people, diabetes age-adjusted comparative prevalence 4.0%, adults with undiagnosed diabetes 19 800 and diabetes-related deaths 544 [1].

By International Expert Committee report on the use of the HbA_1c_ assay in the diagnosis of diabetes, diabetes should be diagnosed when HbA_1c_ is ≥6.5% (48 mmol/mol). Individuals with an HbA_1c_ level ≥6% (42 mmol/mol) but ˂6.5% (48 mmol/mol) are at the highest risk for progression to diabetes [31].

In our study, most HbA_1c_ values were within the limits of the reference value 4.8%–5.9% (29–42 mmol/mol)); 5.8% of cases had values indicating diabetes risk and 3.8% with diabetes mellitus. At the same time, we saw that the higher the age the more frequently the HbA_1c_ value is ≥6.0% (42 mmol/mol). As majority of the deceased were young and middle-aged males and no females were included in our study group, therefore, prevalence of diabetes and diabetes risk was found lower compared to population-based study.

Chen et al. [32] have found that hyperglycaemia was frequently detected in mechanical asphyxiation, saltwater drowning, cerebrovascular disease, ischemic heart disease and electrocution. In deaths caused by external causes we found high HbA_1c_ values only in individual cases, all with concomitant cardiovascular diseases; therefore, it is reasonable to think that the increase of HbA_1c_ is caused by the diseases, not external causes. Although the relationship between cardiovascular disease and glycaemia is believed to represent a continuum without a threshold effect, HbA_1c_ might offer more advantages in terms of prognostic information, as it is a more stable, accurate parameter of glucose homeostasis.

Persistent elevations in blood sugar (therefore HbA_1c_) increase the risk of long-term vascular complications of diabetes such as coronary disease, heart attack, stroke, heart failure, kidney failure, etc. [33,34]. HbA_1c_ is a good marker of glycated proteins, which play a contributory role in atherosclerosis in both diabetic and non-diabetic individuals. We also achieved the similar result, as the HbA_1c_ values were in range 4.3%–10.7% (24–93 mmol/mol), median 5.6% (38 mmol/mol) occurred in 87 cases with cardiovascular diseases, an HbA_1c_ value ≥6.0% (42 mmol/mol) occurred with ischaemic disease, atherosclerosis of aorta and coronary arteries (33.4%) and when the HbA_1c_ value was ≥6.5% (48 mmol/mol), 58% of cases included cardiovascular diseases. Our study also showed that the increase in HbA_1c_ value 6.0%–6.4% (42–46 mmol/mol) occurs together with concomitant affecting cardiovascular diseases: atherosclerosis of the aorta and coronary arteries, hypertension and dilating cardiomyopathy.

Among adults per capita alcohol consumption (in litres of pure alcohol) by type of alcoholic beverage was 41% beer, 37% spirits, 11% wine and 11% other in 2010 [35]. The “other alcohol” group contains surrogate non-beverage alcohols (ethanol-containing liquids not intended for consumption), which are drunk particularly among men in Estonia [36,37]. In a previous study of association of alcohol with the mortality of Estonian men aged 25–54 years, alcohol was found in blood in concentrations ≥ 0.2 mg/g in 55% cases of death [38]. Results of that study of men also showed evidence of alcohol-induced pathologies in 75% of cases, and 32% had pathologies in two or more organs [39]. Some types of alcohol-related purchases were associated with a lower prevalence of type 2 diabetes (T2D), and respondents who purchased the greatest volumes of wine or beer – but not liquor – were less likely to report being diagnosed with T2D in 2011–2012 than non-drinkers [40].

We found a negative correlation between alcohol intoxication and HbA_1c_ value (*r_s_* = −0.1, *P* < 0.05), as deceased in the acute alcohol intoxication group were relatively younger. The effects may be also caused by alcohol-induced hypoglycaemia [41]. In the whole study group 54.6% of the deceased ethanol was determined in blood.

Lower-than-expected levels of HbA_1c_ can be seen in people with shortened red blood cell lifespan, such as with glucose-6-phosphate dehydrogenase deficiency, sickle-cell disease or any other condition causing premature red blood cell death. A low HbA_1c_ value occurred with carbon monoxide poisoning (2.4%) which may be associated with the ability of haemoglobin to bind carbon monoxide.

In the case of poisoning by narcotic substances there are different opinions in the literature about HbA_1c_ values, e.g. according to Chen et al. [32] the HbA_1c_ value is lower in fatal methamphetamine abuse, probably from the starvation during the long-term abusing of methamphetamine. Palmiere et al. [15] showed that cocaine consumption provokes hyperglycaemia due to increases of adrenalin/noradrenalin level. Probably the HbA_1c_ normal values found on the basis of this study may be explained by the fact that the fentanyl typically used in Estonia [42]. Fentanyl does not affect glucose metabolism or that mostly young people die as a result of poisoning with narcotic substances.

The liver is an accumulator of glycogen and a regulator of glucose metabolism. Therefore when liver damage worsens, glucose metabolism is also impaired and the HbA_1c_ level increases. With an HbA_1c_ value ≥6.0% (42 mmol/mol), 35.6% had focal liver steatosis, 28.9% had diffuse steatosis, 4.5% cirrhosis and 2.2% alcoholic liver damage. Liver damage was absent in 24.2% of cases. With an HbA_1c_ value ≥6.0% (42 mmol/mol) of pancreas damage acute pancreatitis occurred in 2.2% and chronic pancreatitis in 15.6% of cases. At the same time we know that autolysis makes the assessment of morphological findings of the pancreas difficult. Therefore, we see every day that it is not possible to assess pancreas findings.

The increasing trends in alcoholic liver cirrhosis mortality in Estonia over the past two decades represent trends for chronic liver disease and cirrhosis in northern European countries [43]. Drastic reduction of the level of liver enzymes ALT and AST in the case of cirrhotic liver damage indicates liver failure in live subjects, but on the basis of these reference values it is not possible to find associations of ALT and AST with cause of death groups, as the same reference ranges cannot be used in deceased subjects. At the same time there was a positive correlation between ALT (and the enzyme associated more with liver damage than AST) and HbA_1c_ in cause of death groups. An AST to ALT ratio over 2:1, characteristic to alcohol abuse, also occurred in our study in alcoholic liver disease groups. In the study by Ringmets et al. [38] it was stated that liver enzymes, used widely to detect liver damage in living subjects, have a limited postmortem value. AST and ALT are increased by haemolysis and hypoxia and so are subject to differences in the process of dying. GGT, as a marker of liver fibrosis and cirrhosis, is less affected by these processes.

When determining the cause of death, only the GGT result of liver enzymes can be used to determine the cause of death (e.g. cirrhotic liver, chronical alcohol abuse). A strong dose-response relationship of serum GGT to cardiovascular mortality has been found [44]. The association with high GGT was consistent over all subgroups of disease with the exception of acute/subacute forms of coronary heart disease in men. However, this latter association was significant in men aged <60 years. Additionally, GGT was significantly associated with established risk factors such as triglycerides, body mass index and cholesterol. Adjustment for these variables in multiple risk factor regression analyses confirmed GGT as an independent predictive factor for cardiovascular mortality.

However, the results in postmortem biochemistry in general do not have an absolute value, and they must be interpreted in the context of pathological findings and the circumstances of the death, and with information from multiple marker systems, to maximize their diagnostic value.

## Conclusion

Since these diabetic complications are often involved in the causes of sudden death or contributors to other casualties, postmortem diagnosis of diabetes mellitus is important in routine forensic autopsy. HbA_1c_ in blood is useful to investigate not only fatal metabolic disorders involved in diabetes mellitus but also death processes due to other causes.

## References

[1] International Diabetes Federation Diabetes atlas. 8th ed Brussels: International Diabetes Federation; 2017 [cited 2018 Jan 12] Available from: http://www.diabetesatlas.org/

[2] Koppes LL, Decker JM, Hendriks HF, et al. Moderate alcohol consumption lowers the risk of type 2 diabetes. A meta-analysis of prospective observational studies. Diabetes Care. 2005;28:719–725. 15735217 10.2337/diacare.28.3.719

[3] Carlsson S, Hammar N, Grill V Alcohol consumption and type 2 diabetes. Meta-analysis of epidemiological studies indicates a U-shaped relationship. Diabetologia. 2005;48:1051–1054. 15864527 10.1007/s00125-005-1768-5

[4] Go VLW, Gukovskaya A, Pandol SJ Alcohol and pancreatic cancer. Alcohol. 2005;35:205–211. 16054982 10.1016/j.alcohol.2005.03.010

[5] Cullmann M, Hilding A, Östenson CG Alcohol consumption and risk of pre-diabetes and type 2 diabetes development in a Swedish population. Diabet Med. 2012;29:441–452. 21916972 10.1111/j.1464-5491.2011.03450.x

[6] Goullé JP, Lacroix C, Bouige D Glycated hemoglobin: a useful post-mortem reference marker in determining diabetes. Forensic Sci Int. 2002;128:44–49. 12208021 10.1016/s0379-0738(02)00152-4

[7] Keltanen T, Sajantila A, Valonen T, et al. HbA_1c_ method evaluation for postmortem samples. Forensic Sci Med Pathol. 2015;11:35–39. 25537421 10.1007/s12024-014-9638-4

[8] Palmiere C Postmortem diagnosis of diabetes mellitus and its complications. Croat Med J. 2015;56:181–193. 26088843 10.3325/cmj.2015.56.181 PMC4500977

[9] American Diabetes Association Diagnosis and classification of diabetes mellitus. Diabetes Care. 2010;33:S62–S69 [cited 2017 Sept 8] Available from: 10.2337/dc10-S062 20042775 PMC2797383

[10] Nathan DM, Turgeon H, Regan S Relationship between glycated haemoglobin levels and mean glucose levels over time. Diabetologia. 2007;50:2239–2244. 17851648 10.1007/s00125-007-0803-0 PMC8752566

[11] Bunn HF, Haney DN, Kamin S, et al. The biosynthesis of human hemoglobin A1c. Slow glycosylation of hemoglobin in vivo. J Clin Invest. 1976;57:1652–1659. 932199 10.1172/JCI108436 PMC436825

[12] Inada M, Oishi M, Nishikawa M, et al. Clinical evaluation of measuring glycosylated hemoglobin levels for assessing the long-term blood glucose control in diabetics. Endocrinol Japan. 1980;27:411–415. 6780328 10.1507/endocrj1954.27.411

[13] Keltanen T, Sajantila A, Valonen T, et al. Measuring postmortem glycated hemoglobin – a comparison of three methods. Leg Med. 2013;15:72–78. 10.1016/j.legalmed.2012.09.002 23089141

[14] Hockenhull J, Dhillo W, Andrews R, et al. Investigation of markers to indicate and distinguish death due to alcoholic ketoacidosis, diabetic ketoacidosis and hyperosmolar hyperglycemic state using post-mortem samples. Forensic Sci Int. 2012;214:142–147. 21840144 10.1016/j.forsciint.2011.07.040

[15] Palmiere C, Bardy D, Mangin P, et al. Postmortem diagnosis of unsuspected diabetes mellitus. Forensic Sci Int. 2013;226:160–167. 23357227 10.1016/j.forsciint.2013.01.004

[16] Brinkmann B, Fechner G, Karger B, et al. Ketoacidosis and lactic acidosis – frequent causes of death in chronic alcoholics? Int J Legal Med. 1998;111:115–119. 9587792 10.1007/s004140050130

[17] Kernbach-Wighton G Diagnostic problems with functional causes of death: analytical approaches and procedures. Leg Med. 2009;11:S31–S35. 10.1016/j.legalmed.2009.01.075 19269226

[18] Chen C, Glagov S, Mako M, et al. Post-mortem glycosylated hemoglobin (HbAlc): evidence diabetes mellitus. Ann Clin Lab Sci. 1983;13:407–410. 6638930

[19] Hindle EJ, Rostron GM, Gatt JA The diagnostic value of glycated haemoglobin levels in post-mortem blood. Ann Clin Biochem. 1985;22:144–147. 4004104 10.1177/000456328502200206

[20] Uemura K, Shintani-Ishida K, Saka K, et al. Biochemical blood markers and sampling sites in forensic autopsy. J Forensic Leg Med. 2008;15:312–317. 18511006 10.1016/j.jflm.2007.12.003

[21] Siigur U, Kallion K, editors. Ühendlabori käsiraamat IV [Handbook of united laboratories IV]. Tartu: Sihtasutus Tartu Ülikooli Kliinikum; 2012. Estonian.

[22] Krug H, Zschoch H Reflexphotometrische Untersuchungen zur Gelbfärbung des Schädeldaches bei Diabetes mellitus. Virchows Arch Pathol Anat Physiol Klin Med. 1964;338:166–171. 14332922

[23] Schafer AT The colour of the human skull. Forensic Sci Int. 2001;117:53–56. 11230946 10.1016/s0379-0738(00)00448-5

[24] Schäfer T, Klintschar M, Lichtinghagen R, et al. Xanthochromia of the skull bone associated with HbA_1c_. Forensic Sci Int. 2016;260:54–58. 26799427 10.1016/j.forsciint.2015.12.030

[25] Tessier FJ The Maillard reaction in the human body. The main discoveries and factors that affect glycation. Pathol Biol. 2010;58:214–219. 19896783 10.1016/j.patbio.2009.09.014

[26] Hess C, Wöllner K, Musshoff F, et al. Detection of diabetic metabolism disorders post-mortem – forensic case reports on cause of death hyperglycaemia. Drug Test Anal. 2013;5:795–801. 23620079 10.1002/dta.1479

[27] Püschel K, Bajanowski T, Vennemann M, et al. Plötzliche und unerwartete Todesfälle aus innerer Ursache. In: Madea B, editor. Rechtsmedizin – Befunderhebung, Rekonstruktion, Begutachtung. Berlin Heidelberg: Springer-Verlag; 2015 p. 445–446.

[28] Hess C, Musshoff F, Madea B Disorders of glucose metabolism–post mortem analyses in forensic cases: part I. Int J Legal Med. 2011;125:163–170. 20878417 10.1007/s00414-010-0509-6

[29] Hess C, Stratmann B, Quester W, et al. Clinical and forensic examinations of glycemic marker 1,5-anhydroglucitol by means of high performance liquid chromatography tandem mass spectrometry. Forensic Sci Int. 2012;222:132–136. 22749674 10.1016/j.forsciint.2012.05.010

[30] Takata T, Yamasaki Y, Kitao T, et al. Measurement of postmortem 1,5-anhydroglucitol in vitreous humor for forensic diagnosis. J Forensic Sci. 2016;61:S150–S153. 26418332 10.1111/1556-4029.12963

[31] The International Expert Committee International expert committee report on the role of the A1c assay in the diagnosis of diabetes. Diabetes Care. 2009;32:1327–1334 [cited 2017 Sept 8] Available from: 10.2337/dc09-9033 19502545 PMC2699715

[32] Chen JH, Michiue T, Inamori-Kawamoto O, et al. Comprehensive investigation of postmortem glucose levels in blood and body fluids with regard to the cause of death in forensic autopsy cases. Leg Med. 2015;17:475–482. 10.1016/j.legalmed.2015.08.004 26593993

[33] Okrainec K, Banerjee DK, Eisenberg MJ Coronary artery disease in the developing world. Am Heart J. 2004;148:7–15. 15215786 10.1016/j.ahj.2003.11.027

[34] Kozakova M, Palombo C Diabetes mellitus, arterial wall, and cardiovascular risk assessment. Int J Environ Res Public Health. 2016;13:201. 26861377 10.3390/ijerph13020201 PMC4772221

[35] Global Information system on alcohol and health (GISAH). World Health Organization. 2014 [cited 2017 Sept 8] Available from: http://www.who.int/substance_abuse/publications/global_alcohol_report/profiles/est.pdf?ua=1

[36] Pärna K, Lang K, Raju K, et al. A rapid situation assessment of the market for surrogate and illegal alcohols in Tallinn: Estonia. Int J Public Health. 2007;52:402–410. 18369003 10.1007/s00038-007-6112-z

[37] Pärna K, Leon DA Surrogate alcohol drinking in Estonia. Alcohol Clin Exp Res. 2011;31:1454–1457. 10.1111/j.1530-0277.2011.01481.x 21463339

[38] Ringmets I, Tuusov J, Lang K, et al. Alcohol and premature death in Estonian men: a study of forensic autopsies using novel biomarkers and proxy informants. BMC Public Health. 2012;12:146. 22369510 10.1186/1471-2458-12-146 PMC3328271

[39] Tuusov J, Lang K, Väli M, et al. Prevalence of alcohol-related pathologies at autopsy: Estonian forensic study of alcohol and premature death. Addiction. 2014;109:2018–2026. 25066373 10.1111/add.12695 PMC4241049

[40] Adjemian MK, Volpe RJ, Adjemian J Relationships between diet, alcohol preference, and heart disease and type 2 diabetes among Americans. PLoS One. 2015;10:e0124351. 25961601 10.1371/journal.pone.0124351 PMC4427330

[41] Musshoff F, Hess C, Madea B, et al. Verkehrsmedizin. In: Madea B, editor. Rechtsmedizin – Befunderhebung, Rekonstruktion, Begutachtung. Berlin Heidelberg: Springer-Verlag; 2015 p. 762.

[42] Tuusov J, Vals K, Tõnisson M, et al. Fatal poisoning in Estonia 2000–2009. Trends in illegal drug-related deaths. J Forensic Leg Med. 2013;20:51–56. 23217376 10.1016/j.jflm.2012.04.023

[43] Pärna K, Rahu K Dramatic increase in alcoholic liver cirrhosis mortality in Estonia in 1992–2008. Alcohol Alcohol. 2010;45:548–551. 20729528 10.1093/alcalc/agq050

[44] Ruttmann E, Brant LJ, Concin H, et al. The Vorarlberg health monitoring and promotion program study group. γ-Glutamyltransferase as a risk factor for cardiovascular disease mortality. An epidemiological investigation in a cohort of 163 944 Austrian adults. Circulation. 2005;112:2130–2137. 16186419 10.1161/CIRCULATIONAHA.105.552547

